# Environmental Difference and Spatial Distance Affect the Fidelity of Variation Source of Microbial Community Structure in Air-Dried Soils

**DOI:** 10.3390/microorganisms10040672

**Published:** 2022-03-22

**Authors:** Zhiying Guo, Yuanyuan Bao, Jie Liu

**Affiliations:** 1 State Key Laboratory of Soil and Sustainable Agriculture, Institute of Soil Science, Chinese Academy of Sciences, Nanjing 210008, China; yybao@issas.ac.cn (Y.B.); liujie@issas.ac.cn (J.L.); 2 College of Advanced Agricultural Sciences, University of Chinese Academy of Sciences, Beijing 100049, China

**Keywords:** soil drying, microbial ecology, spatial scale

## Abstract

Air-dried soil archives are important for microbial ecology research, although the process of air-drying preservation inevitably destroys the original microbial information in soils. Only upon fully understanding the limitations of air-dried soil can it play a greater role. The value of air-dried soil depends on the fidelity of microbial community structure information in the air-dried soil relative to that in fresh soil. To evaluate this, high-throughput sequencing was applied to investigate the microbial community of fresh soils and 227 days air-dried archives from typical farmland under a large spatial scale, and PERMANOVA was used to analyze the explanation proportion (EP) of the spatial factor on the microbial community structure in any paired-fresh or air-dried soils. The results show that for any paired soils, the value of EP ranged from 42.4% to 97.9% (*p* < 0.001). Importantly, taking fresh soil as a reference, the value of EP declined in air-dried soils (effect size *r* = 0.79, *p* < 0.001). Furthermore, the standardized difference in EP between fresh and air-dried soil (NDEP) was used to characterize the fidelity of variance source of microbial community structure in air-dried soils, and correlation tests showed that NDEP was negatively correlated with spatial distance (*r* = −0.21, *p* < 0.01) and with environmental difference (*r* = −0.37, *p* < 0.001). Further analyses show that larger NDEP was observed at a spatial distance <25 km or an environmental difference <0.58. Variance partitioning analysis showed that 28.0% of the variation in NDEP could be explained, with environmental difference constituting 14.0% and the interaction between the environmental difference and spatial distance constituting the remaining 14.0%. Soil texture was the most important factor for predicting NDEP, followed by soil pH and annual average temperature. This study not only emphasizes the possible decline in EP when using air-dried soils to reveal microbial community patterns, but also implies that air-dried soil is more suitable for addressing scientific questions under a large spatial scale or environmental differences.

## 1. Introduction

As an invaluable record of soil quality, many worldwide institutions and organizations (e.g., Rothamsted, NEON and CERN) systematically curate collections of archived soils [1]. These archived soils can be used not only to check and verify the recorded soil information, but also to trace the historical events and ecological environment marks that have not been well documented. These archives are a readily available source of ecological information relevant to microbial ecology, but only when we are clear about the application boundary of air-dried soil archives.

In recent years, many studies have shown that air-dried soil archives play an important role in revealing spatial distribution patterns and community assembly mechanisms, and in tracing environmental changes and the effects of farmland fertilization [2,3,4,5]. Among them, studies on the evaluation of air-dried soil highlighted its value in microbial ecology; however, they overlooked the limitations of such application. They can be summarized in three aspects. Firstly, more attention was focused on whether the distribution and clustering patterns of the microbial community were consistent with expectations rather than on its driving factors and the reproducibility and fidelity of explanation proportions after air-drying preservation. Secondly, in terms of experimental materials, few studies had strict fresh soil samples to act as controls, let alone soil samples of a spatial gradient. Thirdly, lacking quantitative descriptions, most evaluation results were only qualitative. Obviously, air-drying and archiving changed the structure of the soil microbial community, because air-drying indeed influences the in situ and/or original information held by the microbial community due to desiccation [6,7,8,9]. Therefore, the limitations of air-dried soil archives should be fully recognized before use.

**Figure 1:**
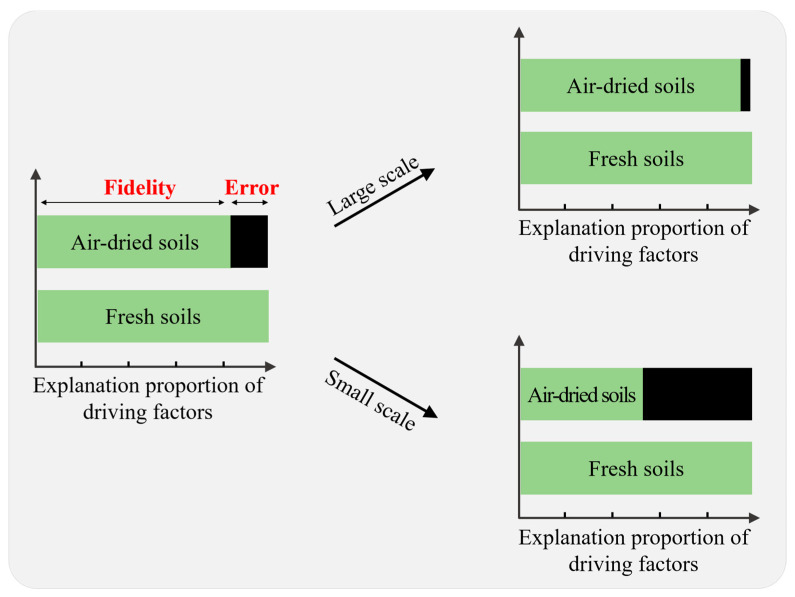
The concept diagram of the changing fidelity of the source of variation in the microbial community structure in the air-dried soils. It is assumed that the microbial community of fresh soils is governed by driving factors such as spatial factors, fertilization management, etc. After the soil is air-dried, the variance source of the community structure of air-dried soils deviates from that of fresh soils. The degree of deviation varies with the scale of soil samples. In short, when the soil samples are vastly different (e.g., a large spatial distance of thousands of kilometers or an environmental difference represented by acid soil vs. alkaline soil), air-drying counts for a small fraction of the original variance source. In this situation, the microbial community structure of air-dried soils would be consistent with that of fresh soil. If the difference between soil samples is small, the air-drying factor counts for a larger fraction of the variance source rendering the community structure of air-dried soils inconsistent with that of fresh soils.

Disturbance is a common way to affect the composition and spatial distribution pattern of the microbial community [10]. The strength of the influence of a disturbance on microbial community composition depends on spatial scales, presenting a greater influence at the local scale and a relatively limited influence at large scales, e.g., regional or continental [11,12]. On a large scale, external disturbance generally cannot hide the differences in microbial communities; in these situations, strong environmental gradients select distinct bacterial species [13]. On a smaller scale, the external disturbance could potentially subvert the microbial community structure because the microbial community generally exhibits a relatively smaller and unstable difference [14]. Used it as an analogy to understand the air-dried soil archives, air-dried preservation is a disturbance that would probably count for a portion of the true source of the variation in the microbial community structure in fresh soils, leading to the distortion of its analysis in air-dried soils. Therefore, it is supposed that the distortion displays a scale effect, such that the variation source losses might be lesser at large scales, and greater at small scales (Figure 1).

To determine whether the source of variation in the microbial structure in air-dried soil changes compared with that in fresh soils and its relationship with scales (i.e., spatial distance and environmental difference), high-throughput sequencing was applied to investigate the bacterial community of fresh and air-dried soils from typical farmland with a spatial span of approximately 2453 km. With fresh soil as a reference, the fidelity and influencing factors of the source of variation in the microbial community structure were quantized in air-dried soil archives. This study refined the application of air-dried soils and enhanced the confidence of applying air-dried soil archives for microbial ecology studies by confirming the changing fidelity of source of variation in the microbial community structure in air-dried soil samples and, the relationship between fidelity and scale.

## 2. Materials and Methods

### 2.1. Soil Sampling

**Figure 2:**
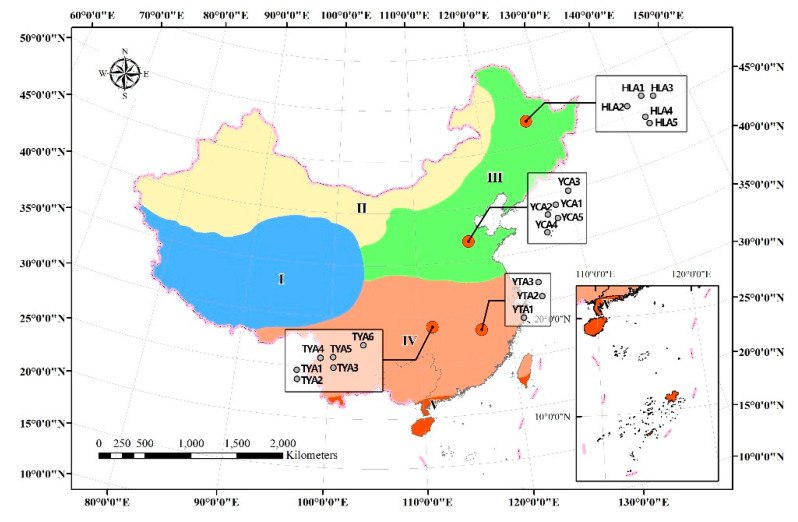
Map of sampling sites in this study. Sampling sites are labeled with red circles and sampling plot names are labeled around sites. The map presents the distribution of five climate zones namely region I to region V, representing the plateau and mountain climate zone, temperate continental climate zone, temperate monsoon climate zone, subtropical monsoon climate zone and tropical monsoon climate zone, respectively.

Four agricultural sites, namely, Hailun (HLA), Yucheng (YCA), Taoyuan (TYA) and Yingtan (YTA) were selected to represent typical farmland in different eco-climate regions of China, and a total of 19 plots (5, 5, 6 and 3 at each site, respectively) were selected for sample collection (Figure 2). The crops of the four sites in the sampling year were rice at YTA, rapeseed at TYA, and corn at YCA and HLA. The distance between sites ranged from 534 km to 2453 km. The distance between plots at each site ranged from meters to 25 km. The surface soil (0–20 cm) inside each plot was collected using the five-point mixing sampling method and plant roots, insects and other non-soil were removed during sampling. After sampling, the soil samples were sent to the laboratory on ice in time. Each soil sample was divided into two parts: one part for air-drying and archiving and the other for the determination of physical and chemical properties. During air-drying and archiving, the soil on day 0 and day 227 was used as fresh soils and air-dried soils, respectively. In total, 38 soil samples were used for microbial community investigation.

### 2.2. Environmental Variables Determination

The soil pH, soil organic carbon (SOC), total nitrogen (TN), total phosphorus (TP), total potassium (TK), texture and water content (WC) were determined, according to standardized methods [11] (Table S1). The annual average temperature (AAT) and annual precipitation (AP) were generated from WorldClim Climatology according to the spatial locations of each plot [15].

### 2.3. DNA Extraction and High-Throughput Sequencing

Genomic DNA was extracted from 0.5 g of soil using a FastDNA SPIN Kit for soil (MP Biomedicals, Santa Ana, CA, USA). The extracted DNA was dissolved in 100 μL of DES buffer. The DNA quantity and quality were assessed using a NanoDrop 2000 (ThermoFisher, Waltham, MA, USA) and the solution was stored at −20 °C for further usage.

The soil DNA was amplified using the universal primers 515F (5′-GTGCCAGCMGCCGCGG-3′) and 907R (5′-CCGTCAATTCMTTTRAGTTT-3′) [16]. A unique barcode sequence (11 mer) was added to the 5′-end of the forward strand, which was then used for the generation of PCR amplicons. The PCR cycling conditions were 94 °C, 5.0 min; 28 × (94 °C, 30 s; 54 °C, 30 s; 72 °C, 45 s); 72 °C, 8 min. A 50 µL PCR reaction mixture containing 45 µL L^−1^ Platinum PCR SuperMix (Invitrogen, Shanghai, China), a 200 nM final concentration of each primer, and 2 µL of template DNA was used; the amplicons were purified using the QIAquick PCR Purification kit (Qiagen, Valencia, CA, USA), and quantified using NanoDrop ND-2000 (Thermo Scientific, Waltham, MA, USA).

All the samples were pooled together with equal molar amounts from each sample. High-throughput sequencing was performed with the Illumina Miseq sequencing platform (Illumina Inc., San Diego, CA, USA). The bar-coded PCR products from all the samples were normalized in equimolar amounts before sequencing.

### 2.4. Bioinformatic Analysis

After sequencing was completed, 16S rRNA gene data were processed using the Quantitative Insights Into Microbial Ecology (QIIME, version 1.9) pipeline for data sets [17] (http://qiime.org, accessed date 1 January 2022). Sequences were assigned to soil samples based on the unique barcode. The paired-end read data were merged using FLASH v1.2.11 [18]. Sequences with an average quality score below 25 and length shorter than 200 bp were trimmed and then the resultant high-quality sequences were binned into OTUs using a 97% identity threshold with the UPARSE algorithm [19]. Simultaneously, chimeras were checked and eliminated. Taxonomic classification was then assigned to OTUs with the RDP database (Trainset No. 16) using a naïve RDP classifier at a confidence threshold of 0.7 [20]. For the evaluation of the beta diversities of soil bacterial phylotypes, samples were rarefied to 40,000 sequences. Sequence data were deposited in the DDBJ DRA database (accession No., PRJDB12506)

### 2.5. Statistical Analysis

Statistical analyses were performed in R v4.0.4 [21]. Between any two soils, soil sampling location (namely, the spatial factor), which can represent geolocation and environmental variables, is the main driving factor of microbial community structure. To evaluate the possible change in the explanation proportions of driving factor (EP) on microbial community structure using air-dried soil, compared with that of fresh soils, the Permutation Multivariate Analysis of Variance (PERMANOVA) test [22] was conducted between any two of 19 fresh or air-dried soils, respectively. The obtained R square value was used to compare the EP in fresh and air-dried soils. PERMANOVA was conducted based on Bray–Curtis distance using the R package “VEGAN” [23]. A schematic overview of the main procedures is given in Figure S1. In detail, firstly, the 171 × 2 groups of paired soils were separately generated from 19 fresh soils and 19 air-dried soils. Secondly, PERMANOVA was performed on each of the 171 × 2 groups with the soil sampling location as the independent variable. Six technical replications were performed by randomly sampling the community table, due to limited samples when performing PERMANOVA. In this step, the EPs of the 171 fresh soil groups ($EP_{fresh}^{1}$,$\;EP_{fresh}^{2}$, $EP_{fresh}^{3}$, …, $EP_{fresh}^{171}$) and the EPs of the 171 air-dried soil groups ($EP_{air-dried}^{1}$,$\;EP_{air-dried}^{2}$, $EP_{freair-dried}^{3}$, … ,$\;EP_{freair-dried}^{171}$) were obtained through PERMANOVA tests. These two series of EPs were matched, and the paired Wilcoxon rank-sum test was used to compare the difference in EP; the distribution of the changes ($EP_{fresh}-EP_{air-dried}$) was also plotted. Thirdly, for the matched fresh and air-dried group, the normalized difference explanation proportion (NDEP) was calculated using the formula below. It was used here as a parameter to assess the degree of fidelity of the microbial community information in air-dried soils.

$$NDEP^{i}=\frac{EP_{fresh}^{i}-EP_{air-dried}^{i}}{EP_{fresh}^{i}}$$

where, *i* is the group id of ranging from 1 to 171, and $NDEP^{i}$, $EP_{fresh}^{i}$, $EP_{air-dried}^{i}$ are the normalized difference explanation proportion, the EP of fresh soils, and the EP of air-dried soils in group *i*, respectively.

The changing NDEP was linked to the background difference of soil samples, including spatial distance and environmental difference. The spatial distance was calculated based on the latitudes and longitudes of each soil using the R package “geodist”. Environmental difference was calculated using the Euclidean distance [24] of all the variables in the ecological context including physical and chemical parameters of the soil, and climate variables. Before calculation, each variable was normalized to dimensionless form using the method “Min-Max”. The relationship between NDEPs and the background difference was evaluated using the Spearman method. Moreover, to clearly visualize the changing pattern of NDEP across spatial and environmental differences, the continuous data sets of spatial distance and environmental distance were broken into a series of scale breaks according to Jenks’ natural break method using the “plotJenks” function in the “GmAMisc” package. Meanwhile, the variation partitioning analysis (VPA) was performed to distinguish the spatial and environmental influence on NDEP using the “varpart” function of the “VEGAN” package.

To evaluate the relative importance of the environmental variable difference on influencing NDEP, we used a model selection based on an exhaustive search method to select the best predictors of NDEP. This procedure was performed using the function ‘regsubsets’ in the R package “leaps”. All predictors were standardized before analyses using the “Min-Max” method to interpret parameter estimates on a comparable scale. The relative effect of the parameter estimates for each of the predictors was compared with the effect of all parameter estimates in the model. 

To test which specific OTUs changed significantly during soil storage, differential abundance analysis between fresh soils and air-dried soils was performed on the relative abundance of OTUs using the Wilcoxon signed-rank test following the *p* value correction (Bonferroni correction method). Considering the large differences in microbial communities across samples, only those OTUs that appeared in more than 90% of the samples were analyzed (192 OTUs, the sum of relative abundance of these OTUs across samples ranged from 8.45% to 27.87%, with a mean value of 14.64%).

## 3. Results

### 3.1. General Description of Soil Microbial Community Composition

Based on high-throughput sequencing, a total of 5,111,383 high-quality sequences were obtained from 38 soil samples. The sequencing depth of each sample was normalized to 45,379 reads and 33,474 OTUs were identified at 97% similarity. The relative abundance of 10 most dominant taxa in all the soil samples was investigated at the phylum level (Figure S2). The most dominant phyla across all the soil samples were Proteobacteria (11.2–32.2%), Chloroflexi (6.2–46.5%), Actinobacteria (4.7–28.5%), Acidobacteria (2.9–26.5%), Planctomycetes (3.6–16.8%), Firmicutes (0.8–40.2%), Bacteroidetes (0.1–8.7%), Gemmatimonadetes (0.1–5.9%), Crenarchaeota (0.2–5.2%) and Nitrospirae (0.1–2.9%). These phyla accounted for more than 90% of the total sequences in all soil samples (96.2–96.5% at HLA, 92.0–93.1% at TYA, 95.5–95.9% at YCA and 91.0–93.8% at YTA). The members of these dominant phyla remained unchanged in the air-dried state compared with that under fresh state at four sites.

### 3.2. Decline of EP in Air-Dried Soils Compared with That in Fresh Soils

**Figure 3:**
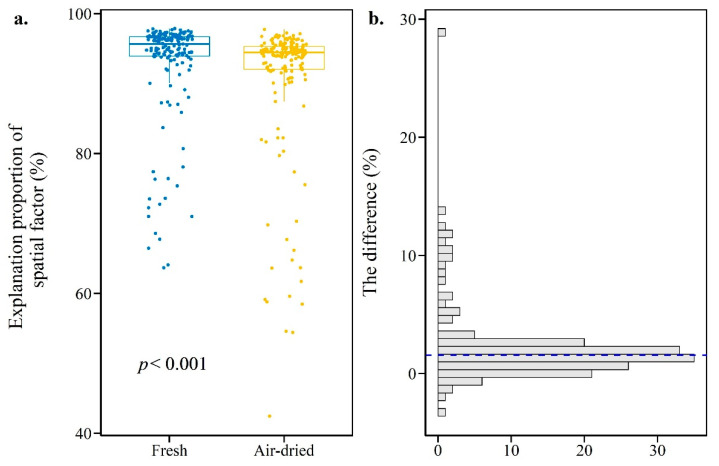
The proportion of explanation for the spatial factor which drives the microbial community structure in fresh soils (blue) and air-dried soils (yellow) (**a**). The distribution of the difference in the proportion of explanation for the spatial factor between fresh soils and air-dried soils. The x-axis represents frequency, and the blue dash line represents median value (**b**).

The PERMANOVA test showed that for all 342 groups of paired soils (171 fresh soils and 171 air-dried soils), the proportion of explanation for the spatial factor (EP) on microbial community structure measured with R square ranged from 42.4% to 97.9% (*p* < 0.001) (Table S2). Figure 3a showed that the median EP in the fresh group was 95.6% (IQR = 2.74%), whereas the median in the air-dried group was 94.4% (IQR = 3.25%). The Wilcoxon test showed that the difference was significant (*p* < 0.001, effect size *r* = 0.79). Figure 3b showed that the difference in EP between fresh and air-dried soil ranged from −3.6% to 28.6% with a median value of 1.6%. It should be noted that among 171 comparisons, 89.5% of them had a declining EP from fresh to air-dried soils.

### 3.3. NDEP Varies with Spatial Distance and Environmental Difference

**Figure 4:**
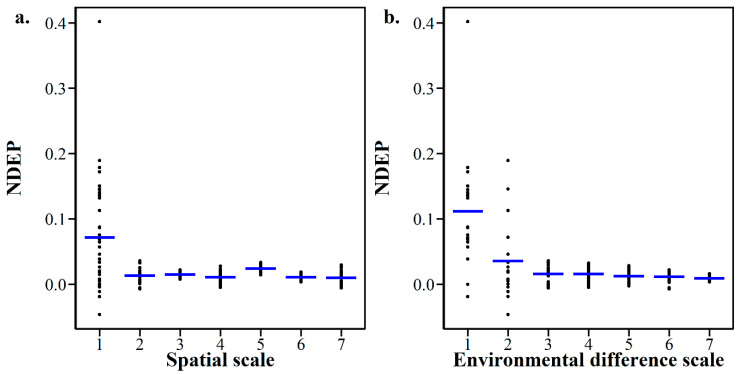
NDEP varies with spatial scale (**a**) and environmental difference scale (**b**). The blue line in the box represents the median value.

To make data comparable, the difference in EP in fresh and air-dried soils was standardized with NDEP. The results show that NDEP ranged from −0.048 to 0.402 with an average of 0.031 and a median value of 0.017. The Spearman correlation test showed that NDEP was negatively correlated with spatial distance (*r* = −0.21, *p* < 0.01) and environmental difference (*r* = −0.37, *p* < 0.001) (Figure S3). For better visualization, Jenks’ natural break method was applied to break the values of spatial distance and environmental difference into seven different classes: the larger the value, the larger the scale. Figure 4 shows that NDEP tended to be larger at smaller scales and smaller at larger scales, whether under the spatial scale or environmental difference scale (Figure 4a,b). To be specific, NDEP was significantly higher at spatial scale 1 than other scales with median and average values of 0.061 and 0.072, respectively, and higher at environmental difference scale 1 than others with median and average values of 0.088 and 0.112, respectively (Table 1). 

**Figure 5:**
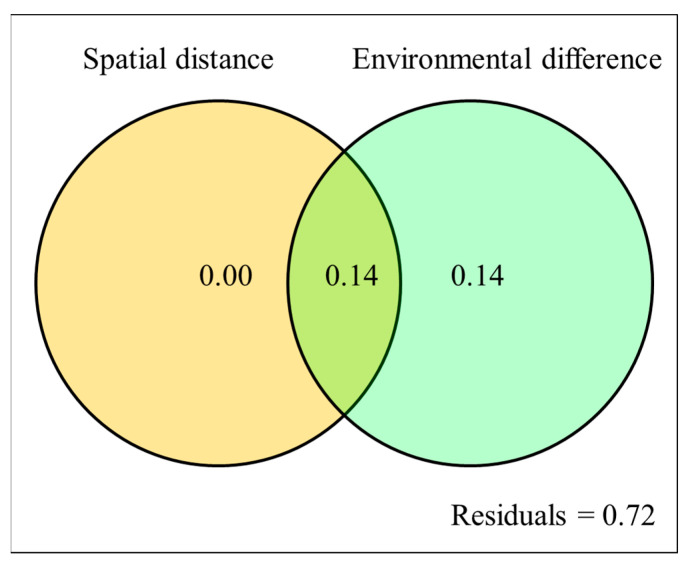
The contribution of spatial distance and environmental difference to NDEP based on Variance Partitioning Analysis. The left yellow circle represents spatial distance, and the right green circle represents environmental difference.

Variance partitioning analysis (VPA) was used to evaluate the effects of the spatial scale and environmental differences scale on NDEP. Figure 5 shows that 28.0% of the variation in NDEP could be explained, with environmental difference constituting 14.0% and interaction between environmental difference and spatial distance constituting the other14.0%. The large part of the variation (72%) cannot be explained by either environmental difference or spatial distance.

### 3.4. The Main Environmental Variables Influencing NDEP

The relative influence of the environmental variable difference on NDEP was further evaluated. The results show that the estimates of all the significant environmental variables were negative, and the sand content was the most important variable (30.66%, *p* < 0.01) for NDEP prediction, followed by the clay content (24.31%, *p* < 0.05), annual average temperature (23.67%, *p* < 0.01) and soil pH (21.36%, *p* < 0.05) (Table 2). In other words, soil properties could explain 76.33% of the variation in NDEP, and of all soil properties, the relative importance of physical properties (54.97%) was larger than that of chemical properties (21.36%).

### 3.5. OTUs Differential Abundance Analysis between Fresh Soils and Air-Dried Soils

**Figure 6:**
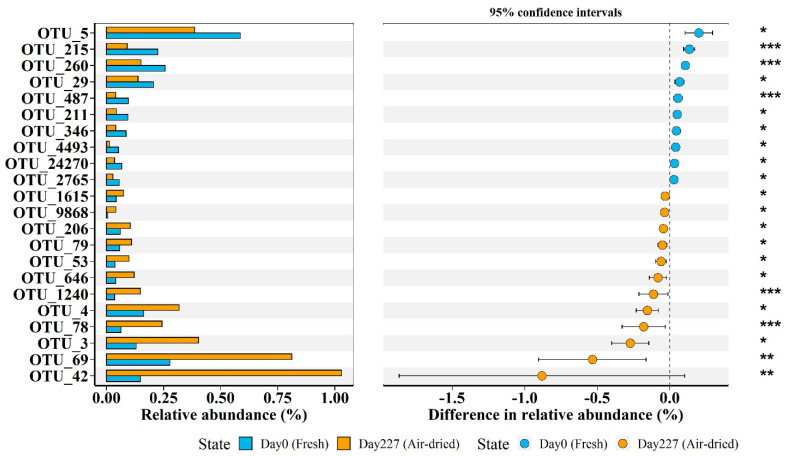
Extended error bar plot identifying significant differences between mean relative abundance of OTU taxa on treatment Day 0 (Fresh) (blue) and treatment Day 227 (Air-dried) (yellow). The symbols on the right indicate the significant *p* value. *** represents *p* < 0.001, ** represents *p* < 0.01, * represents *p* < 0.05. The taxonomic assignment of these significantly changed OTUs is provided in Table S5.

The Wilcoxon signed-rank test following the *p* value correction was used to determine which specific OTUs changed significantly between fresh soils and air-dried soils. A table of the OTUs used in this study is provided in Table S4. Figure 6 shows that 10 OTUs (including OTU_1615, OTU_9868, OTU_206, OTU_79, OTU_53, OTU_646, OTU_1240, OTU_4, OTU_78, OTU_3, OTU_69, and OTU_42) decreased in relative abundance in air-dried soils compared with that in fresh soils. The difference in relative abundance for these OTUs reached a maximum of 0.20%. The other 12 OTUs (including OTU_5, OTU_215, OTU_260, OTU_29, OTU_487, OTU_211, OTU_346, OTU_4493, OTU_24270, and OTU_2765) increased in relative abundance in air-dried soils compared with that in fresh soils. The difference in relative abundance for these OTUs reached a maximum of 0.88%. Most of the OTUs with increasing relative abundance belonged to the Firmicutes phyla except for OTU_646 (Chloroflexi phyla) and OTU_206 (Proteobacteria phyla) (Table S5). The OTUs with decreasing relative abundance belonged to the Proteobacteria and Acidobacteria phyla.

## 4. Discussions

With the change in EP as the core concern and guided by the conceptual diagram (Figure 1), taking the fresh soils of a spatial gradient as reference, this study used indicators such as NDEP to evaluate the fidelity of microbial community information in air-dried soils. The results show that the spatial factor significantly contributed to the variation in the microbial community structure in air-dried soils (Table S2), which is consistent with previous studies [2,3]. More importantly, this study found that the level of EP in air-dried soil declined compared with that in fresh soil (Figure 3). Although this novel insight is proposed here for the first time, a similar phenomenon has also appeared in previous studies, but failed to attract attention. In a recent paper on air-dried soil application, the main driving factor, fertilization, could explain a greater amount of the variance in fresh soils than in air-dried soils [3]. This study suggests that errors in the source of variance caused by air-drying preservation should be seriously considered when solving microbial ecological problems with air-dried soil archives, since analysis of the source of variance is the most common indicator to reveal changes in the microbial community.

NDEP, which characterizes the fidelity in the contribution of the driving factors to the microbial community pattern in air-dried samples, was proved to be negatively correlated with spatial distance, as well as environmental difference. Additionally, as shown above, NDEP started from a relatively high value at limited distance and reached a low value at a relatively large distance (Figure 4 and Figure 5), which confirmed the concept that this study initially expected (Figure 1). Not only did it provide a good explanation for the results of previous studies [2], it also provided a chance for tentative prediction and extrapolation on the changing scale. The higher NDEP at smaller scales of spatial distance or environmental difference questions the over-optimistic assertion that air-drying does not affect the soil microbial community structure when using a DNA-molecular method [25]. At smaller scales, which usually mean a relatively small distance in spatial or other ecological factors, or a specific soil type, soil microbial community pattern information could be influenced by the effect of air-drying and archiving [26,27]. At larger scales, which usually means a spatial large distance or environmental difference, soil microbial community pattern information can be well preserved, and kept largely free from the influence of air-drying and archiving [4,28,29,30,31]. The scale is of significance in governing soil microbial community distribution [32]. On a large scale, the distribution pattern generally exhibits the huge difference in the presence or absence of some microbial species caused by strong environmental gradients, which could are hardly affected by the external disturbance of air-drying and preservation [13]. As shown in Figure S2, the presence of dominant species remains unchanged at the phylum level in air-dried soils compared with that in fresh soils. On a small scale, however, the distribution pattern is probably caused by the difference in the relative abundance of species, which could be easily affected by air-drying preservation. 

In this study, it was found that, of the various environmental variables, only the AAT, clay content, sand content and soil pH had a significant effect on the change in NDEP change. The importance of these variables for microbial community differentiation has been confirmed by many studies [33,34,35]. With a negative coefficient (Table 2), the greater the difference in these variables, the smaller the NDEP, namely, the smaller bias of EP after air-drying and preservation. This is consistent with the concept diagram mentioned above (Figure 1). These results offer practical guidance when the difference in ecological context variables (especially soil texture, soil pH and AAT, etc.) is greater; air-dried soil archives could serve to reveal the distribution pattern of the microbial community.

It is interesting that the relative abundance of OTUs changed consistently after air-drying and preservation, exemplified by the decrease in Proteobacteria OTUs and the increase in Firmicutes OTUs. This is rarely mentioned in previous studies on applicability in the microbial ecology of air-dried soils. This is consistent with the changes in soil microbial composition under drought stress [36,37].

The reasons for using air-drying as the chosen treatment method applied to fresh soil samples in this research are that: first, air-dried soils are currently and widely archived and have been increasingly developed for the study of soil microbial ecology; second, few studies have mentioned the inadequacy of using air-dried samples. Nevertheless, freeze-drying is also a common treatment of biological samples, and its impact on the microbial community requires further study.

## 5. Conclusions

Through investigations of the microbial community and comparisons of the source of variance in the community structure between fresh and air-dried farmland soil samples along a spatial gradient, it is clear that air-dried archives could be applied to reveal microbial community distribution patterns; however, the sources of variance were biased compared with fresh soils, and the fidelity of the variance sources was greater under a larger environmental difference or spatial distance. The environmental variables, including soil texture, soil pH, and are important variables for influencing the fidelity of the source of variance. The significance of this study lies in the further refinement of potential application scenarios for air-dried archives, which are more suitable for solving microbial ecological problems at large scales (i.e., environmental difference and spatial distance). In terms of further study, a more successive spatial gradient (e.g., 25 km to 500 km) should be considered to define a more precise relationship of NDEP with scale.

## Figures and Tables

**Table 1:** Variation in NDEP values at different spatial scales and environmental difference scales.

Spatial Scale	Spatial Distance (km)	Median NDEP Values	Average NDEP Values	Number
1	24.666	0.061	0.072	38
2	541.730	0.014	0.013	18
3	975.764	0.015	0.015	15
4	1020.115	0.012	0.011	30
5	1457.705	0.024	0.024	25
6	2305.852	0.012	0.011	15
7	2452.856	0.007	0.010	30
**Environmental Difference Scale**	**Environmental Difference (Euclidean)**	**Median NDEP Values**	**Average NDEP Values**	**Number**
1	0.577	0.088	0.112	19
2	0.945	0.019	0.036	17
3	1.324	0.018	0.016	27
4	1.535	0.017	0.016	44
5	1.772	0.014	0.012	26
6	2.061	0.013	0.012	26
7	2.450	0.009	0.009	12

**Table 2:** Effect of environmental variable distance on NDEP. See Table S3 for model selection.

	Estimate	Std. Error	t Value	*p* Value	Relative Effect of Estimates
AAT	−0.03925	0.008156	−4.812	*p* < 0.001	23.67%
Sand	−0.05083	0.012719	−3.996	*p* < 0.001	30.66%
Clay	−0.04031	0.015062	−2.676	*p* < 0.05	24.31%
pH	−0.03542	0.010842	−3.267	*p* < 0.05	21.36%

## Data Availability

The sequences were deposited into the DDBJ database accession No., PRJDB12506.

## Supplementary Materials

The following supporting information can be downloaded at: https://www.mdpi.com/article/10.3390/microorganisms10040672/s1. Table S1. Geographic position and environmental variables of soil samples. Table S2. PEMANOVA report of any paired fresh or air-dried soils. Table S3. Results of model selection. Table S4. The OTU table used in this study. Table S5. Taxonomic assignment of OTUs in Figure 6. Figure S1. A schematic overview of the main statistical procedures of this study. Figure S2. The relative abundances of the dominant phyla across all soil samples under two states (i.e., fresh and air-dried) at four sites. Figure S3. The distribution of NDEP along spatial (a) and environmental differences (b). The Spearman method was used to check the relationship between NDEP and spatial distance, as well as environmental difference.

## References

[1] Dolfing J., Feng Y. (2015). The importance of soil archives for microbial ecology. Nat. Rev. Microbiol..

[2] Liu J., Guo Z., Xu A., Wang C., Wu S., Liu Y., Pan K., Zhang F., Pan X. (2019). Consistent spatial distribution patterns of bacterial communities revealed by serial time-archived soils from long-term field experiments. Soil Biol. Biochem..

[3] Wang F., Che R., Deng Y., Wu Y., Tang L., Xu Z., Wang W., Liu H., Cui X. (2021). Air-drying and long time preservation of soil do not significantly impact microbial community composition and structure. Soil Biol. Biochem..

[4] Liang Y., Ning D., Lu Z., Zhang N., Hale L., Wu L., Clark I.M., McGrath S.P., Storkey J., Hirsch P.R. (2020). Century long fertilization reduces stochasticity controlling grassland microbial community succession. Soil Biol. Biochem..

[5] Knapp C.W., Dolfing J., Ehlert P.A., Graham D.W. (2010). Evidence of increasing antibiotic resistance gene abundances in archived soils since 1940. Environ. Sci. Technol..

[6] Potts M. (1994). Desiccation tolerance of prokaryotes. Microbiol. Rev..

[7] Kakumanu M.L., Cantrell C.L., Williams M.A. (2013). Microbial community response to varying magnitudes of desiccation in soil: A test of the osmolyte accumulation hypothesis. Soil Biol. Biochem..

[8] Dadenko E.V., Kazeev K.S., Kolesnikov S.I., Val’kov V.F. (2009). Changes in the enzymatic activity of soil samples upon their storage. Eurasian Soil Sci..

[9] Benucci G.M.N., Rennick B., Bonito G. (2020). Patient propagules: Do soil archives preserve the legacy of fungal and prokaryotic communities?. PLoS ONE.

[10] Ge Y., He J.Z., Zhu Y.G., Zhang J.B., Xu Z., Zhang L.M., Zheng Y.M. (2008). Differences in soil bacterial diversity: Driven by contemporary disturbances or historical contingencies?. ISME J..

[11] Chen R., Zhong L., Jing Z., Guo Z., Li Z., Lin X., Feng Y. (2017). Fertilization decreases compositional variation of paddy bacterial community across geographical gradient. Soil Biol. Biochem..

[12] Deng Y., He Z., Xiong J., Yu H., Xu M., Hobbie S.E., Reich P.B., Schadt C.W., Kent A., Pendall E. (2016). Elevated carbon dioxide accelerates the spatial turnover of soil microbial communities. Glob. Change Biol..

[13] Shi Y., Li Y., Xiang X., Sun R., Yang T., He D., Zhang K., Ni Y., Zhu Y.-G., Adams J.M. (2018). Spatial scale affects the relative role of stochasticity versus determinism in soil bacterial communities in wheat fields across the North China Plain. Microbiome.

[14] Shade A., Jones S.E., Caporaso J.G., Handelsman J., Knight R., Fierer N., Gilbert J.A. (2014). Conditionally Rare Taxa Disproportionately Contribute to Temporal Changes in Microbial Diversity. Mbio.

[15] Fick S.E., Hijmans R.J. (2017). WorldClim 2: New 1-km spatial resolution climate surfaces for global land areas. Int. J. Climatol..

[16] Stubner S. (2002). Enumeration of 16S rDNA of Desulfotomaculum lineage 1 in rice field soil by real-time PCR with SybrGreen (TM) detection. J. Microbiol. Methods.

[17] Caporaso J.G., Kuczynski J., Stombaugh J., Bittinger K., Bushman F.D., Costello E.K., Fierer N., Pena A.G., Goodrich J.K., Gordon J.I. (2010). QIIME allows analysis of high-throughput community sequencing data. Nat. Methods.

[18] Magoč T., Salzberg S.L. (2011). FLASH: Fast length adjustment of short reads to improve genome assemblies. Bioinformatics.

[19] Edgar R.C. (2013). UPARSE: Highly accurate OTU sequences from microbial amplicon reads. Nat. Methods.

[20] Wang Q., Garrity G.M., Tiedje J.M., Cole J.R. (2007). Naive Bayesian classifier for rapid assignment of rRNA sequences into the new bacterial taxonomy. Appl. Environ. Microbiol..

[21] R Core Team (2010). R: A Language and Environment for Statistical Computing.

[22] Anderson M.J. (2017). Permutational Multivariate Analysis of Variance (PERMANOVA). Wiley StatsRef: Statistics Reference Online.

[23] Philip D. (2003). VEGAN, a package of R functions for community ecology. J. Veg. Sci..

[24] Feng Y., Chen R., Stegen J.C., Guo Z., Zhang J., Li Z., Lin X. (2018). Two key features influencing community assembly processes at regional scale: Initial state and degree of change in environmental conditions. Mol. Ecol..

[25] Martí E., Càliz J., Montserrat G., Garau M.A., Cruañas R., Vila X., Sierra J. (2012). Air-drying, cooling and freezing for soil sample storage affects the activity and the microbial communities from two mediterranean soils. Geomicrobiol. J..

[26] Fierer N., Schimel J.P., Holden P.A. (2003). Influence of drying–rewetting frequency on soil bacterial community structure. Microb. Ecol..

[27] Liu Y., Yao H., Huang C. (2009). Assessing the effect of air-drying and storage on microbial biomass and community structure in paddy soils. Plant Soil.

[28] Dolfing J., Vos A., Bloem J., Ehlert P.A.I., Naumova N.B., Kuikman P.J. (2004). Microbial diversity in archived soils. Science.

[29] Clark I.M., Hirsch P.R. (2008). Survival of bacterial DNA and culturable bacteria in archived soils from the Rothamsted Broadbalk experiment. Soil Biol. Biochem..

[30] Macdonald C.A., Ang R., Cordiner S.J., Horswell J. (2011). Discrimination of soils at regional and local levels using bacterial and fungal T-RFLP profiling. J. Forensic Sci..

[31] Liu J., Guo Z., Xu A., Wang C., Wu S., Pan K., Zhang F., Pan X. (2019). Spatial differences in bacterial communities preserved in soils archived for a decade. Appl. Soil Ecol..

[32] Martiny J.B., Eisen J.A., Penn K., Allison S.D., Horner-Devine M.C. (2011). Drivers of bacterial beta-diversity depend on spatial scale. Proc. Natl. Acad. Sci. USA.

[33] Jiao S., Lu Y. (2020). Soil pH and temperature regulate assembly processes of abundant and rare bacterial communities in agricultural ecosystems. Environ. Microbiol..

[34] Tripathi B.M., Stegen J.C., Kim M., Dong K., Adams J.M., Lee Y.K. (2018). Soil pH mediates the balance between stochastic and deterministic assembly of bacteria. ISME J..

[35] Xia Q., Rufty T., Shi W. (2020). Soil microbial diversity and composition: Links to soil texture and associated properties. Soil Biol. Biochem..

[36] Naylor D., DeGraaf S., Purdom E., Coleman-Derr D. (2017). Drought and host selection influence bacterial community dynamics in the grass root microbiome. ISME J..

[37] Pérez Castro S., Cleland E.E., Wagner R., Sawad R.A., Lipson D.A. (2019). Soil microbial responses to drought and exotic plants shift carbon metabolism. ISME J..

