# Supplementary material for: Environmental Difference and Spatial Distance Affect the Fidelity of Variation Source of Microbial Community Structure in Air-Dried Soils
Source: Microorganisms. 2022 Mar 22;10(4):672. doi: 10.3390/microorganisms10040672 (PMC9031423; doi:10.3390/microorganisms10040672)
Supplement: Supplementary file 1 [file microorganisms-10-00672-s001.zip › Figure S1.pdf]

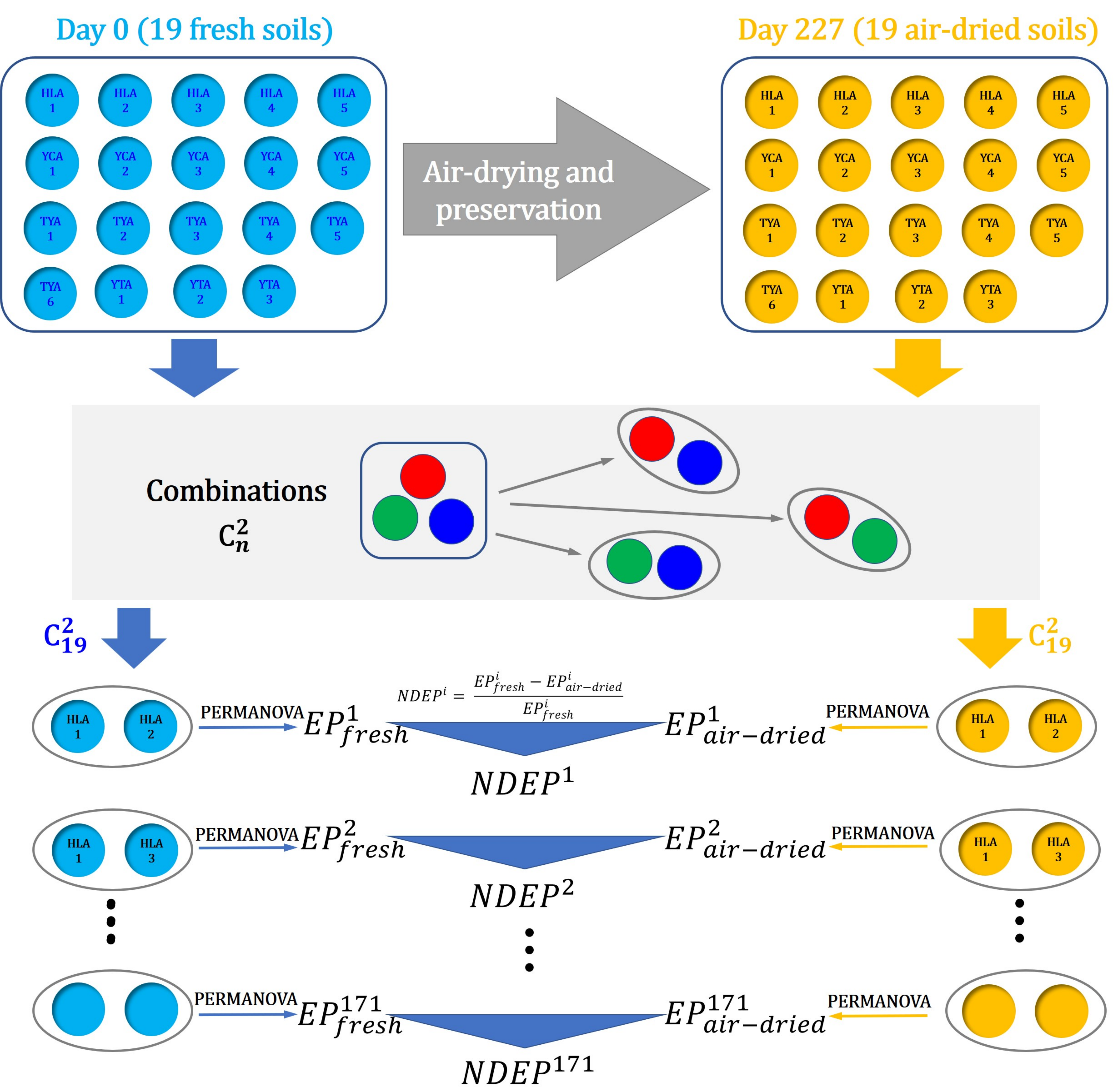

Day 0 (19 fresh soils)
Day 227 (19 air-dried soils)
HLA 1
HLA 2
HLA 3
HLA 4
HLA 5
YCA 1
YCA 2
YCA 3
YCA 4
YCA 5
TYA 1
TYA 2
TYA 3
TYA 4
TYA 5
TYA 6
YTA 1
YTA 2
YTA 3
Air-drying and preservation
Combinations
C_n^2
C_{19}^2
$NDEP^i = \frac{EP^i_{fresh} - EP^i_{air-dried}}{EP^i_{fresh}}$
PERMANOVA
EP^1_{fresh}
$EP^1_{air-dried}$
$NDEP^1$
EP^2_{fresh}
$EP^2_{air-dried}$
$NDEP^2$
EP^{171}_{fresh}
$EP^{171}_{air-dried}$
$NDEP^{171}$
