# Supplementary figures and images for: Environmental Difference and Spatial Distance Affect the Fidelity of Variation Source of Microbial Community Structure in Air-Dried Soils
Source: Microorganisms. 2022 Mar 22;10(4):672. doi: 10.3390/microorganisms10040672 (PMC9031423; doi:10.3390/microorganisms10040672)

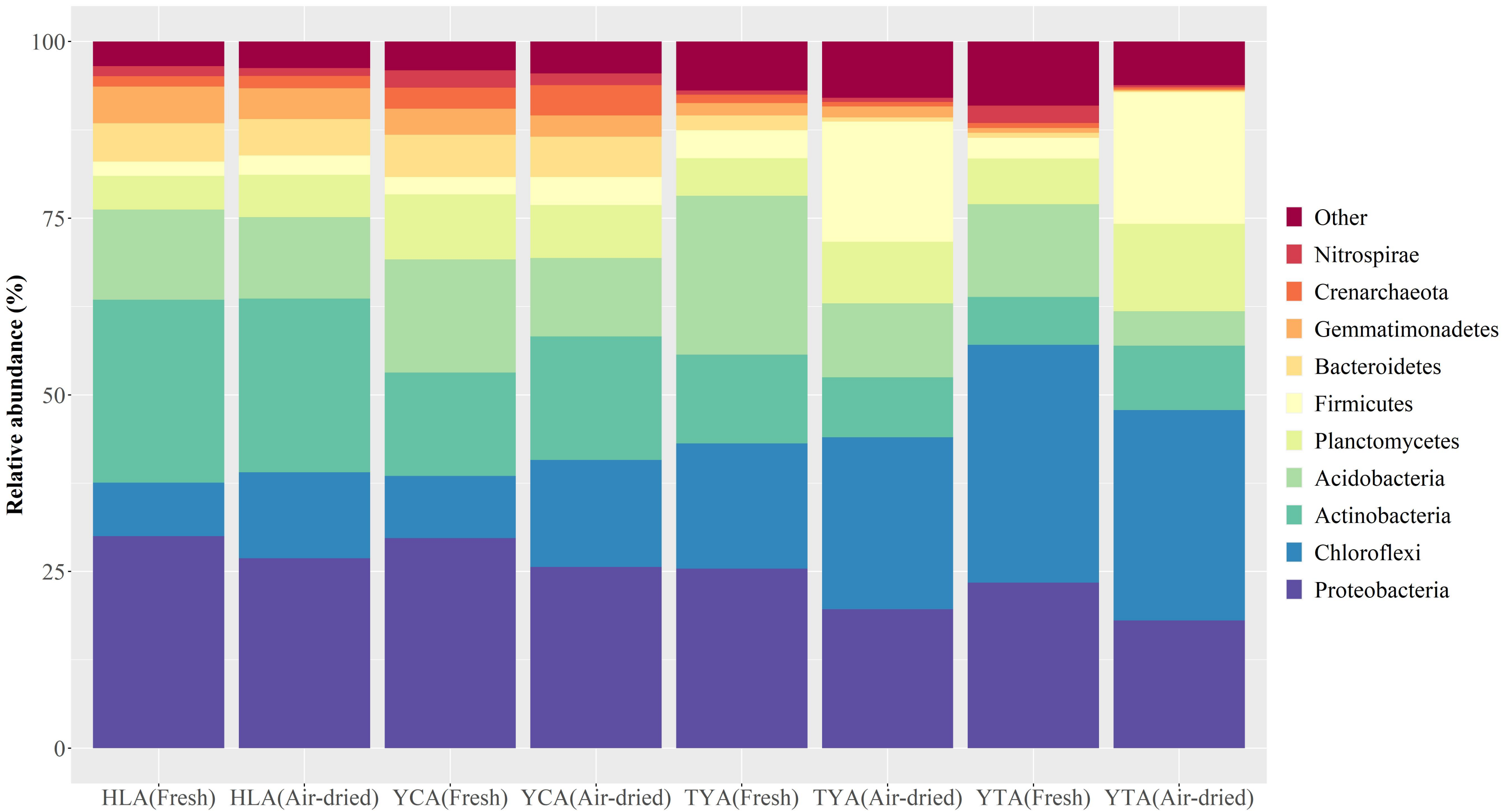

Supplement: Supplementary file 1 [file microorganisms-10-00672-s001.zip › Figure S2.pdf]

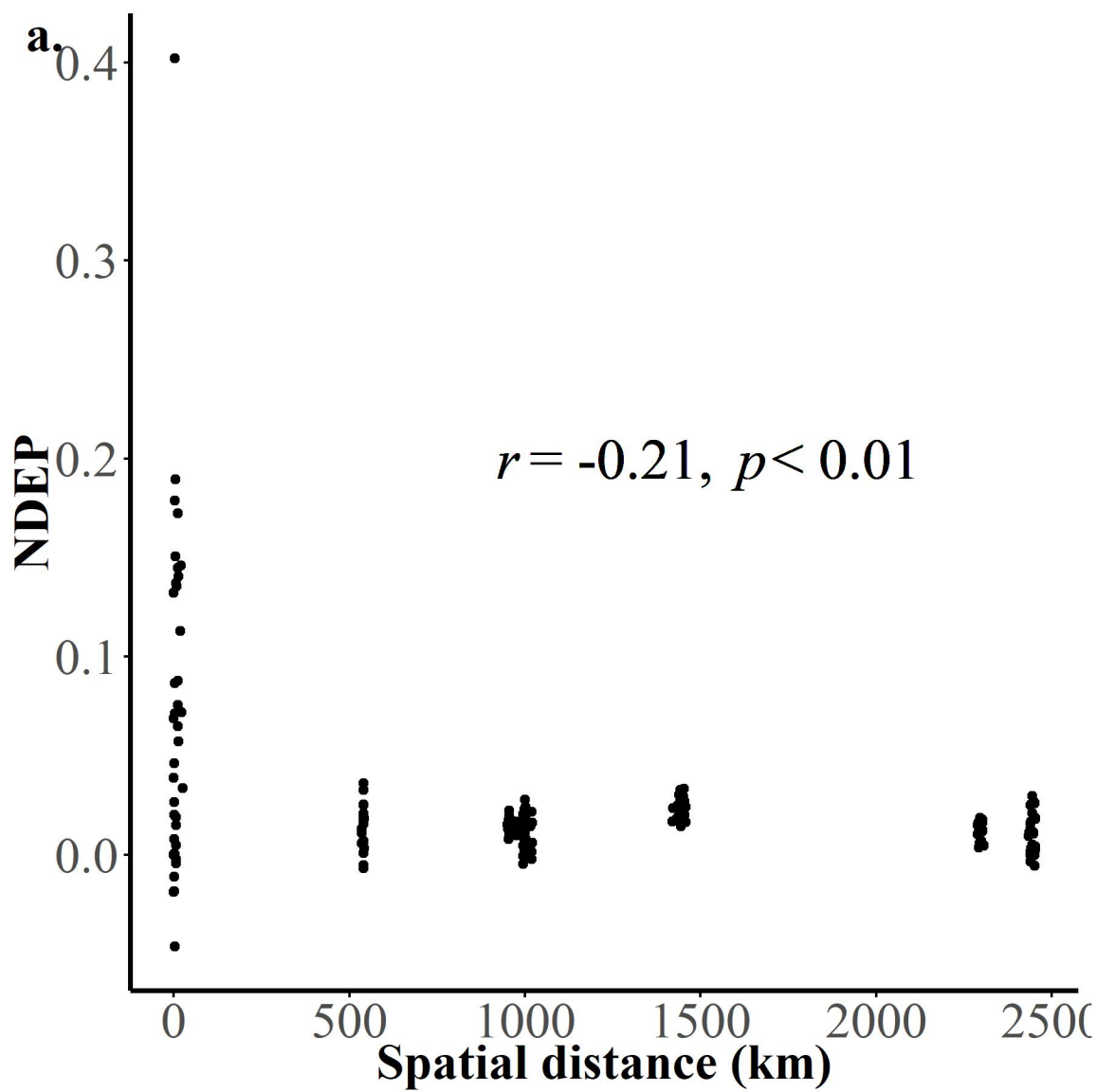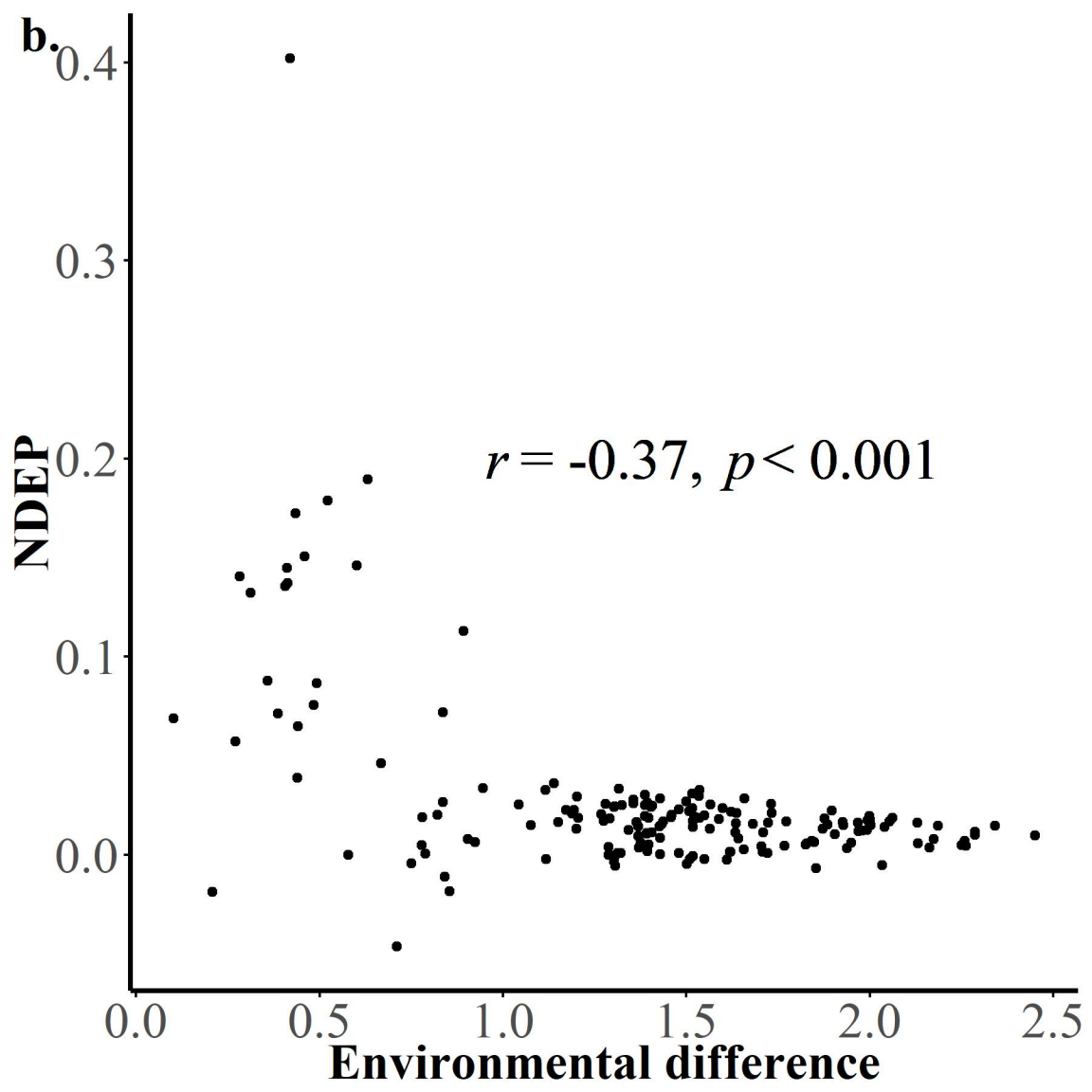

Supplement: Supplementary file 1 [file microorganisms-10-00672-s001.zip › Figure S3.pdf]
